# Supplementary material for: Hepatic TET3 contributes to type-2 diabetes by inducing the HNF4α fetal isoform
Source: Nat Commun. 2020 Jan 17;11:342. doi: 10.1038/s41467-019-14185-z (PMC6969024; doi:10.1038/s41467-019-14185-z)
Supplement: Supplementary file 1 — Supplementary Information [file 41467_2019_14185_MOESM1_ESM.pdf]

SUPPLEMENTARY INFORMATION

Hepatic TET3 contributes to type-2 diabetes by inducing the HNF4α fetal isoform

Li et al.

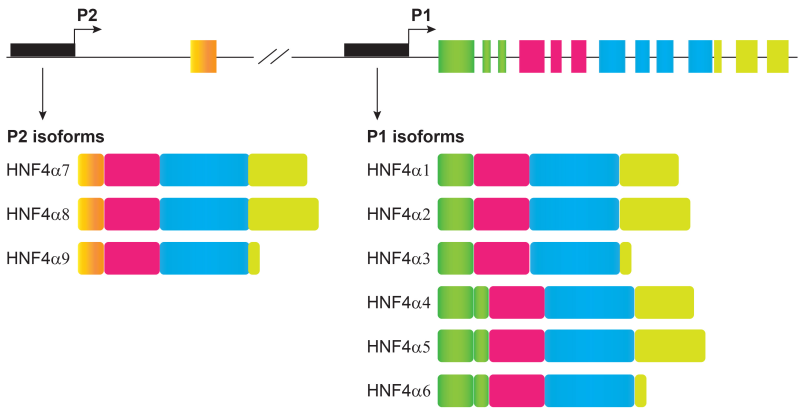

Supplementary Figure 1 Schematic of the HNF4α gene. The gene is composed of 13 exons and contains two promoters, P2 and its downstream P1, that drive expression of 9 isoforms (α1 to α9) via alternative splicing. Antibodies that specifically recognize the N-terminal regions of P2 (orange) and P1 (green) isoforms, respectively, have been well-characterized. siRNAs targeted to the region downstream of P2 and upstream of P1 promoters have been designed to specifically knockdown the P2 isoforms without altering expression of P1 isoforms.

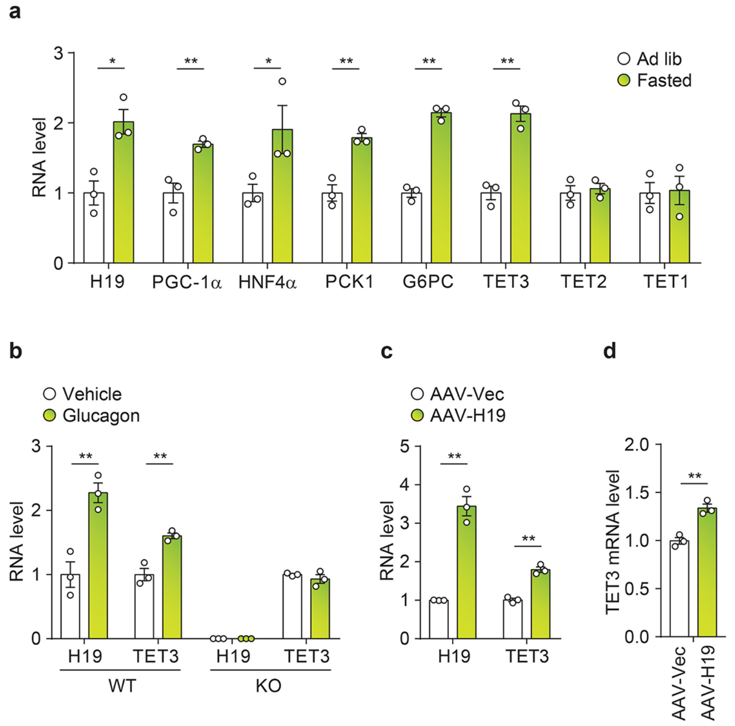

Supplementary Figure 2 Glucagon-induced, H19 mediated upregulation of TET3. **a,** qPCR of the indicated RNAs in liver tissues isolated from mice fed *ad libitium* or fasted for 12 h. n=3. **b,** qPCR of H19 and TET3 RNAs from WT and H19 KO primary hepatocytes treated with vehicle or glucagon (20 nM) for 24 h. n=3. **c,** qPCR of H19 and TET3 RNAs from mouse primary hepatocytes infected with AAV-Vec or AAV-H19 for 48 h. n=3. **d,** qPCR of TET3 mRNA from liver tissues isolated from mice infected with AAV-Vec or AAV-H19 for 14 days. n=3.
Data are representative of two independent experiments and are presented as mean ± SEM. $*p < 0.05$, $**p < 0.01$.

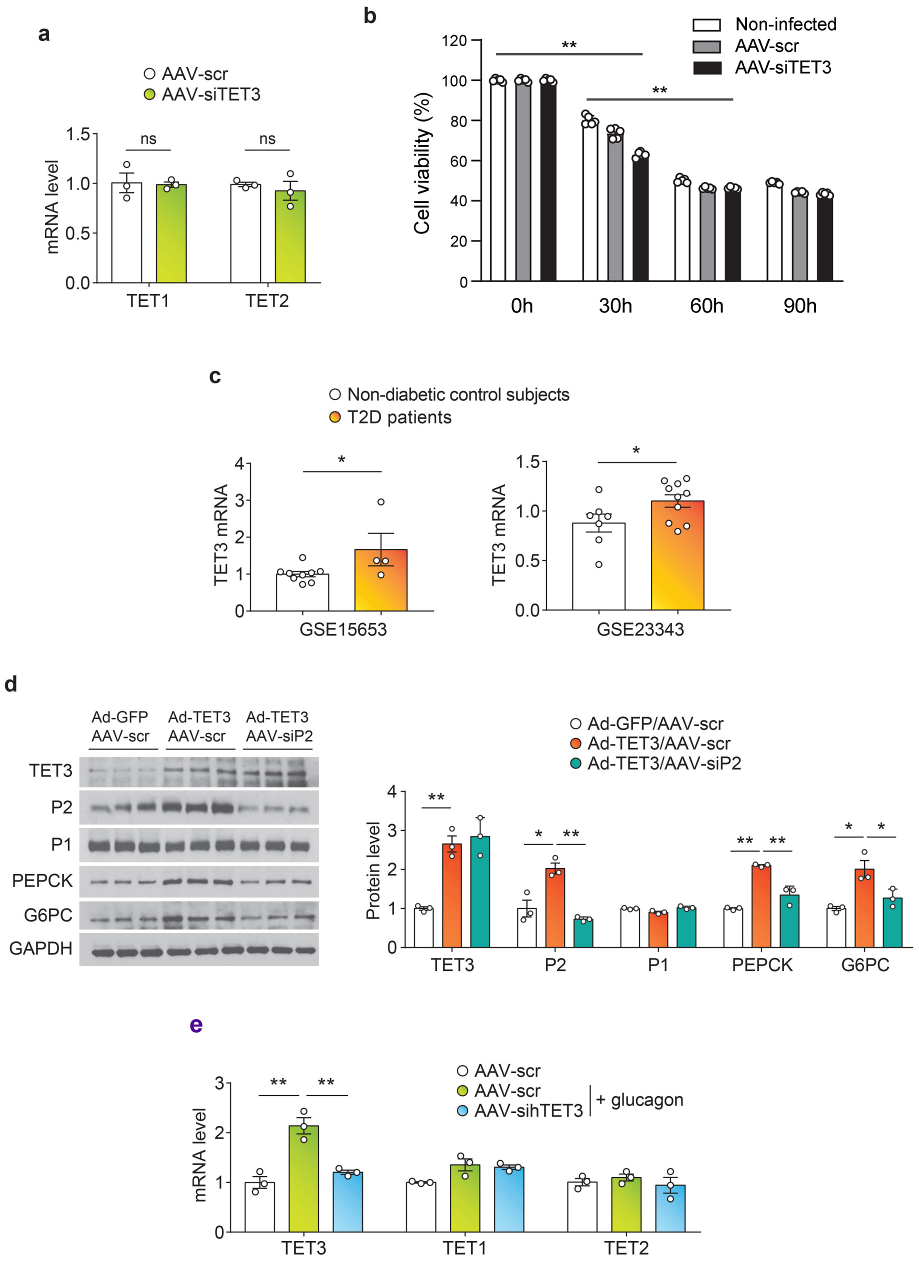

Supplementary Figure 3 TET3 and hepatic gluconeogenic gene expression. **a,** qPCR of TET1 and TET2 from primary mouse hepatocytes infected with AAV-scr or AAV-siTET3. n=3. **b,** Primary mouse hepatocytes were non-infected or infected with AAV-scr or AAV-siTET3 for the indicated time points. Cell viabilities were measured and results are presented as percentage of viabilities of freshly isolated cells (0h). **c,** Hepatic *TET3* expression based on datasets available from the Gene Expression Omnibus (accession numbers GSE15653 and GSE23343). The Robust Rank Aggregation (RRA) method was also used to integrate and analyze the two datasets to obtain integrated differentially expressed TET3 between control and diabetic human subjects to be statistically significant (p=0.01858). **d**, IB of TET3, HNF4α P2 and P1, PEPCK, and 6GPC from H19 KO hepatocytes infected with Ad-GFP plus AAV-scr, Ad-TET3 plus AAV-scr, or Ad-TET3 plus AAV-siP2 for 72 h. n=3, One-way ANOVA with Tukey post-test. **e**, qPCR of TET3, TET2, and TET1 in human primary hepatocytes infected with AAV-scr or AAV-sihTET3 for 48 h and treated with vehicle or glucagon (20 nM) for 24 h. n=3. One-way ANOVA with Tukey post-test.
All data are representative of two independent experiments and are presented as mean ± SEM. $*p < 0.05$, $**p < 0.01$.

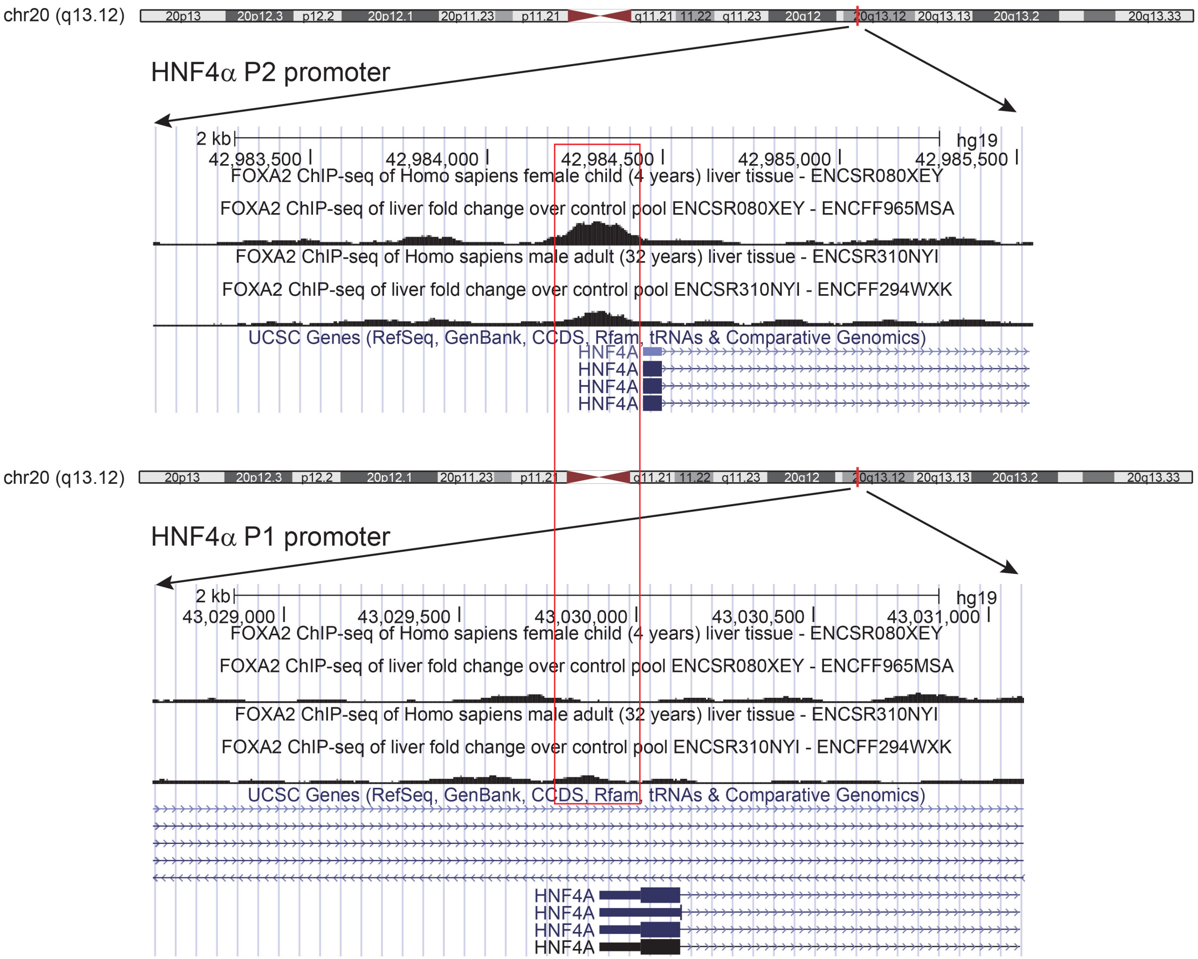

Supplementary Figure 4 Genome browser view showing ChIP-seq of FOXA2 enrichment at HNF4α P2 promoter in human livers. This FOXA2 enrichment region overlaps with the homologous region in the mouse HNF4α P2 promoter (related to Fig. 4a, TET3/FOXA2 ChIP region highlighted in red).

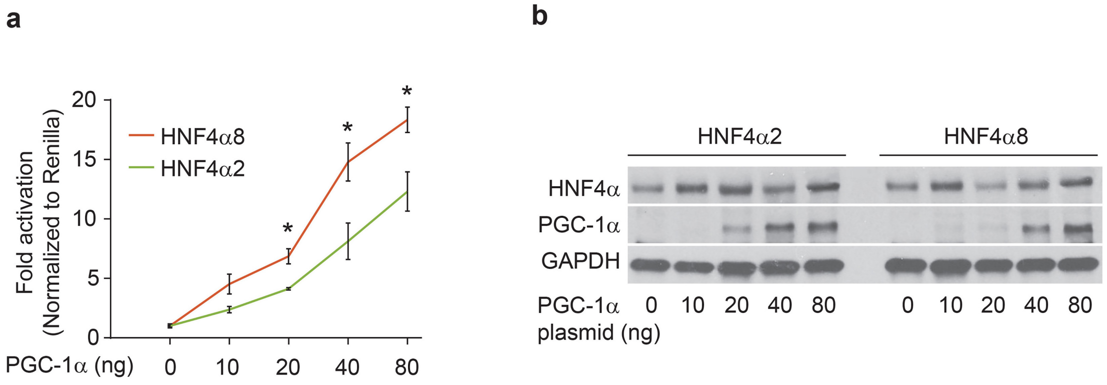

Supplementary Figure 5 Luciferase reporter assays. **a**, U-2 OS cells were transfected with plasmids expressing human HNF4α8 or HNF4α2, together with gAF1, a *Renilla* luciferase reporter, and increasing amounts of a PGC-1α expression vector. Luciferase reporter levels were measured 24 h later. n=3, Two-way ANOVA with Sidak post-test. **b**, IB of HNF4α and PGC-1α from U-2 OS cells treated as in **a**.
Data are representative of two independent experiments and are presented as mean ± SEM. *$p < 0.05$.

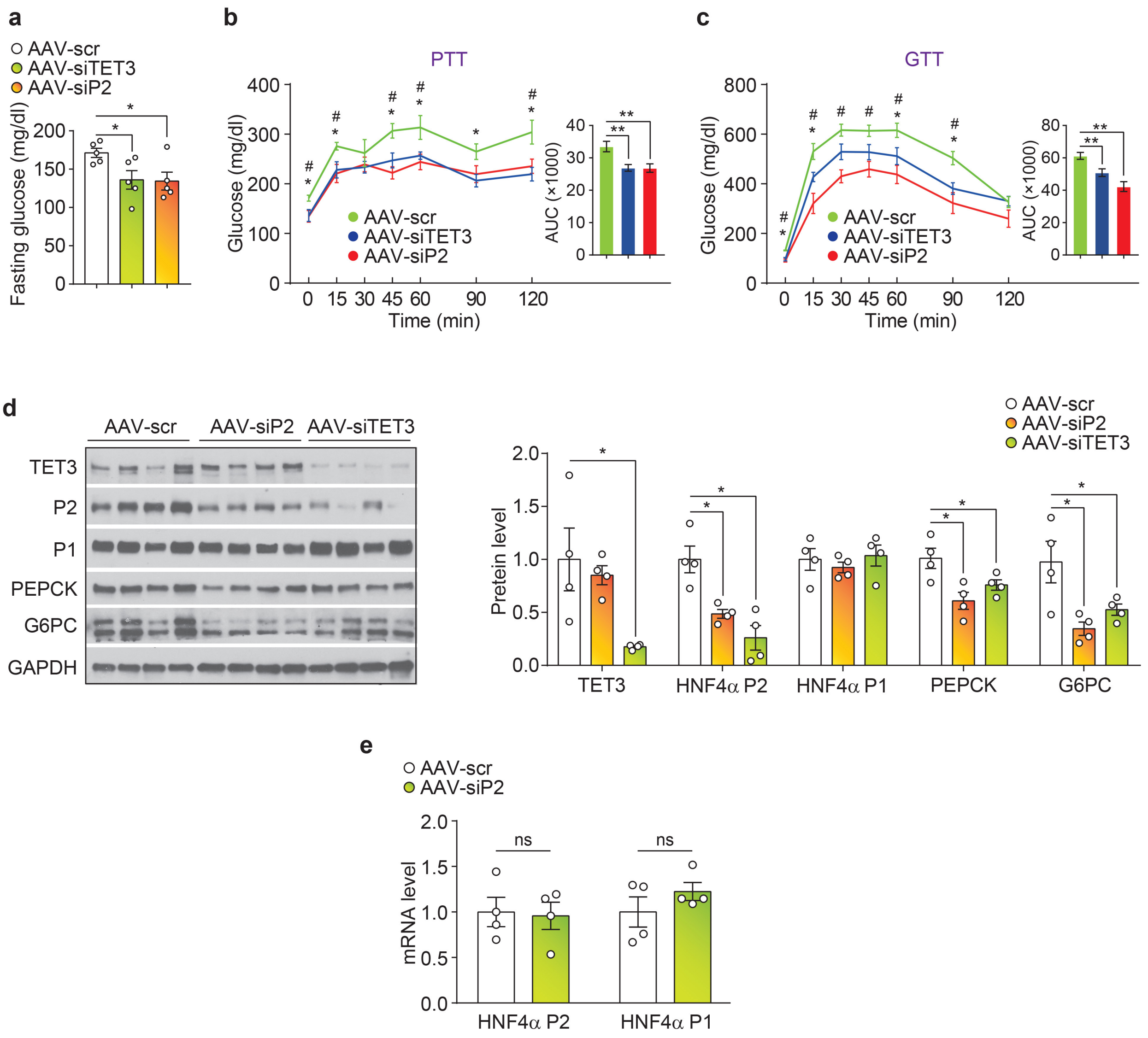

Supplementary Figure 6 TET3-induced P2 isoform contributes to increased HGP in *ob/ob* mice. **a**, Fasting blood glucose in *ob/ob* mice infected with AAV-scr, AAV-siTET3, or AAV-siP2 for 10 days. n=5, One-way ANOVA with Dunnett post-test. **b**, PTT in *ob/ob* mice treated as in **a**. * AAV-siTET3 vs. AAV-scr, # AAV-siP2 vs. AAV-scr. n=5, Two-way ANOVA with Sidak post-test. **c**, GTT in *ob/ob* mice treated as in **a**. n=5, Two-way ANOVA with Sidak post-test. **d**. IB of TET3, HNF4α P2 and P1 isoforms, and PEPCK and G6PC proteins of liver tissues isolated from mice treated as in **a**. n=4, One-way ANOVA with Dunnett post-test. **e**. qPCR of HNF4α P2 and P1 isoforms in pancreas tissues isolated from HFD mice infected with AAV-scr or AAV-siP2 for 10 days. n=4.

Data are representative of two independent experiments and are presented as mean ± SEM. #$p < 0.05$, *$p < 0.05$, **$p < 0.01$. AUC, area under the curve.

Supplementary Table 1

Gene	Forward Primer	Reverse Primer
**Human real-time PCR primer sequences**		
Human HNF4α P2	5’-CTTACGGTCTGCAGTTTCCA-3’	5’-ACATCCTCCTCCTGCTGCTA-3’
Human HNF4α P1	5’-GGTTTGAAAGGAAGGCAGAG-3’	5’-AGGGTGGTGTAGGCTGGGTC-3’
Human TET1	5’-GCAGCGTACAGGCCACCACT-3’	5’- AGCCGGTCGGCCATTGGAAG-3’
Human TET2	5’-TTCGCAGAAGCAGCAGTGAAGAG-3’	5’-AGCCAGAGACAGCGGGATTCCTT-3’
Human TET3	5’-GACGAGAACATCGGCGGCGT-3’	5’-GTGGCAGCGGTTGGGCTTCT-3’
Human PCK1	5’-GGTTCCCAGGGTGCATGAAA-3’	5’-CACGTAGGGTGAATCCGTCAG-3’
Human G6PC	5’-CCTCAGGAATGCCTTCTACG-3’	5’-TCTCCAATCACAGCTACCCA-3’
Human RPLP0	5’-GGCGACCTGGAAGTCCAACT-3’	5’- CCATCAGCACCACAGCCTTC-3’
**Mouse real-time PCR primer sequences**		
Mouse H19	5’-CCTCAAGATGAAAGAAATGGTGCTA-3’	5’-TCAGAACGAGACGGACTTAAAGAA-3’
Mouse PGC-1α	5’-AACCACACCCACAGGATCAGA-3’	5’-TCTTCGCTTTATTGCTCCATGA-3’
Mouse HNF4α	5’-CAGAATGAGCGGGACCGGATC-3’	5’-CAGCAGCTGCTCCTTCATGGAC-3’
Mouse HNF4α P2	5’-GCGAGTCCTTATGCCCTCA-3’	5’-TGAATTGAGGTTGGCACCTT-3’
Mouse HNF4α P1	5’-ATGGGCAATGACACGTCC-3’	5’-CTCACGCTCCTCCTGAAGAA-3’
Mouse PCK1	5’-TGTTTACTGGGAAGGCATCG-3’	5’-AGGTCTACGGCCACCAAAG-3’
Mouse G6PC	5’-ATCCGGGGCATCTACAATG-3’	5’-TGGCAAAGGGTGTAGTGTCA-3’
Mouse RPLP0	5’-GATGGGCAACTGTACCTGACTG-3’	5’-CTGGGCTCCTCTTGGAATG-3’
Mouse TET1	5’-AAGAAGAGGAAATGCGAGGT-3’	5’-GGCCATTTACTGGTTTGTTG-3’
Mouse TET2	5‘-AGCAAGAGATTCCGAAGGAT-3’	5’-AGTGGAGGACTGAGTGCAAG-3’
Mouse TET3	5’-TGCGATTGTGTCGAACAAATAGT-3’	5’-TCCATACCGATCCTCCATGAG-3’
Mouse GAPDH	5’-CCTTCATTGACCTCAACTACAT-3’	5’-CAAAGTTGTCATGGATGACC-3’
**TET3/FOXA2 ChIP-qPCR and hMeDIP primer sequences**		
Mouse HNF4α P2	5’-TGAGGTCTAGCCAGGTTGC-3’	5’-AGTCCACCAGGAAGGCAGT-3’
Mouse HNF4α P1	5’-ATTAGCACCCCAGGTGTCAG-3’	5’-TGTCCTCTGGGAGACTCAGC-3’
**RNAP ChIP-qPCR primer sequences**		
Mouse HNF4α P2	5’-ACTGCCTTCCTGGTGGACT-3’	5’-TCCTCTATCCTCCCCAACCT-3’
Mouse HNF4α P1	5’-GCTGAGTCTCCCAGAGGACA-3’	5’-AAAGACCCGCTCCATACCTC-3’
**QMSP primer sequences**		
Mouse Hnf4a methylated	5’-GATTAGAAGAATTAATAAGATAATCGGGC-3’	5’-AAACAAAAACCCACACACAACAAC-3’
Mouse Hnf4a unmethylated	5’-GTGATTAGAAGAATTAATAAGATAATTGGGTG-3’	5’-AAACAAAAACCCACACACAACAA-3’

Supplementary Table 2

Reagents or Resources	Source	Identifier
**Antibodies**		
Rabbit polyclonal anti-G6PC (1:500)	Abcam	ab83690
Rabbit polyclonal anti-PEPCK (1:1000)	Abcam	ab70358
Rabbit monoclonal anti-GAPDH (1:3000)	Abcam	ab128915
Mouse monoclonal anti-HNF4α P1 (1:1000)	Abcam	ab41898
Mouse monoclonal anti-HNF4α P2 (1:500)	R&D Systems	PP-H6939-00
Rabbit polyclonal anti-TET3 (1:500)	Millipore Sigma	ABE290
Rabbit polyclonal anti-Ser-5(P)-RNAP	Abcam	ab5131
Goat anti-FOXA2 (1:500)	R&D	AF2400
Rabbit IgG	Sigma-Aldrich	PP64B
Goat IgG	Sigma-Aldrich	I5256
**Chemicals**		
Glucagon	Sigma-Aldrich	G2044
Sodium Pyruvate	Sigma-Aldrich	P5280
Sodium lactate	Sigma-Aldrich	71718
L-Glutamine	GIBCO	25030-081
D-(+)-Glucose	Sigma-Aldrich	G5767
Insulin human	Eli Lilly	HI-210(ND1042)
Insulin	GIBCO	12585-014
Dexamethasone	Sigma-Aldrich	D4902
**Critical commercial kits**		
Amplex Red Glucose/Glucose Oxidase Assay	Invitrogen	A22189
Rat/Mouse Insulin ELISA Kit	Millipore Sigma	EZRMI-13K
Dual-Luciferase Reporter Assay	Promega	E1960
Pierce Agarose ChIP Kit	Thermo Scientific	26156
PrimeScript cDNA Reverse Transcription Kit	TaKaRa	RR037A
SYBR Green PCR Master Mix	Bio-Rad	172-5124
miScript II RT Kit	QIAGEN	218161
Lipofectamine™ 3000 Transfection Reagent	Thermo Fisher Scientific	L3000015
PureLink RNA Mini Kit	Ambion	12183018A
Quick-gDNA MicroPrep	Zymo Research Corporation	D3021
EZ DNA Methylation-Gold Kit	Zymo	D5006
hMeDIP Kit	Epigentek	P-1038-48
Goat TrueBlot	Rockland	18-8814-31
Rabbit TrueBlot	Rockland	18-8816-33
**Experimental models: primary cells and cell lines**		
U2OS	Sigma	92022711
Human cryopreserved hepatocytes	Sigma/BioIVT	M00995
**Experimental models: organisms/strains**		
Mouse:B6.Cg-Lepob	The Jackson Laboratory	000632
Mouse:C57BL/6J-DIO	The Jackson Laboratory	380050
**Oligonucleotides**		
ChIP/hMeDIP primers	Table S1	N/A
qPCR primers	Table S1	N/A
QMSP primers	Table S1	N/A
**Recombinant DNA**		
pAd-Track Flag HA PGC-1alpha	(Lerin et al., 2006)	N/A
Renilla-luciferase	(Rodgers et al., 2005)	N/A
gAF1-luciferase	(Rodgers et al., 2005)	N/A
FR_HNF4A8	(Erdmann et al., 2007)	N/A
FR_HNF4A2	(Thomas et al., 2004)	N/A

Fig. 1b

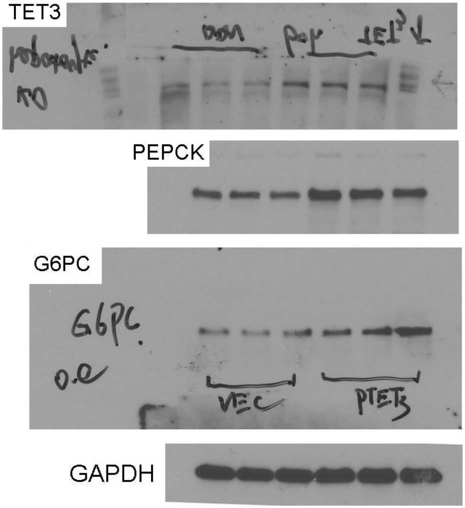

Fig. 1e

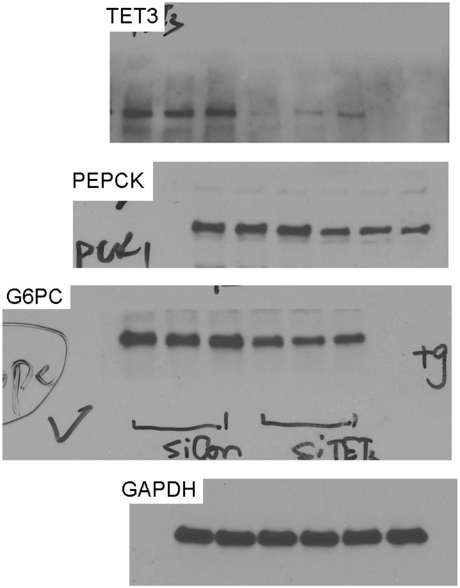

Fig. 1l

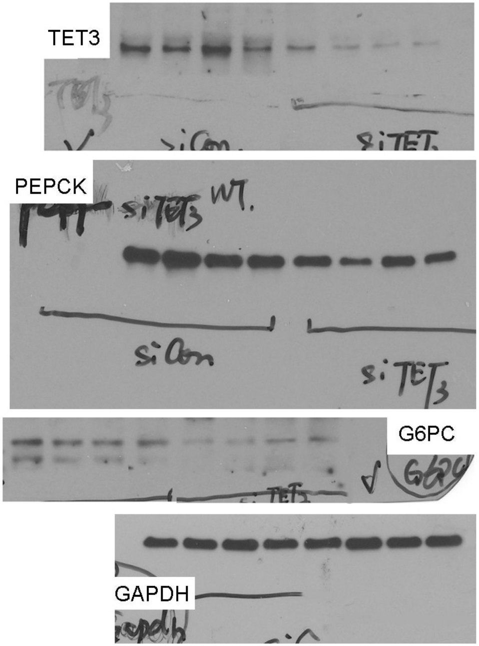

Fig. 1h

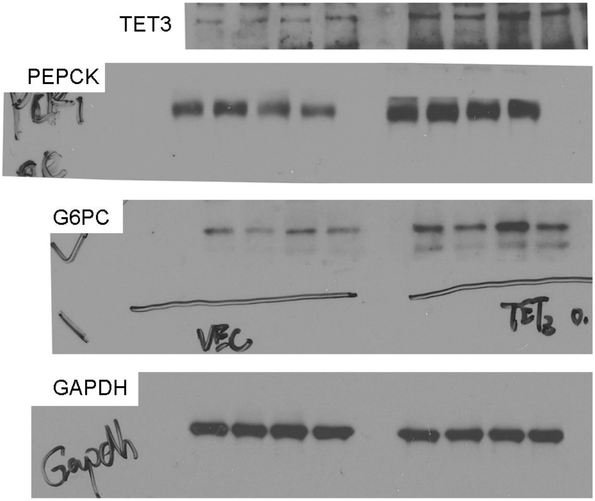

Fig. 2a

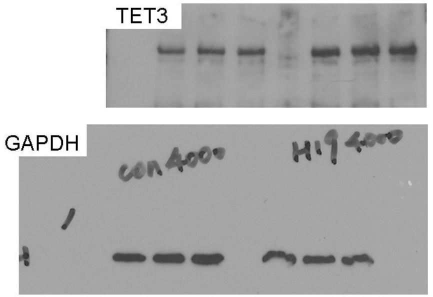

Fig. 2b

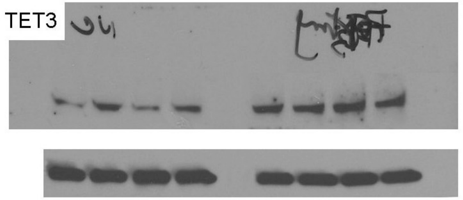

Fig. 2c

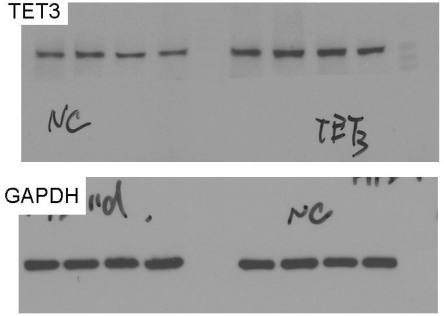

Fig. 2d

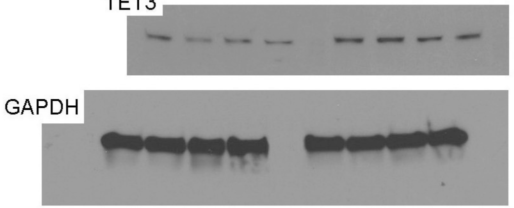

Fig. 2i

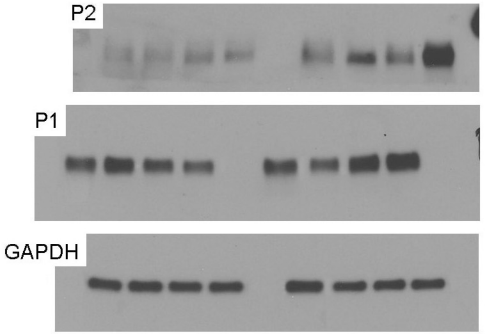

Fig. 2j

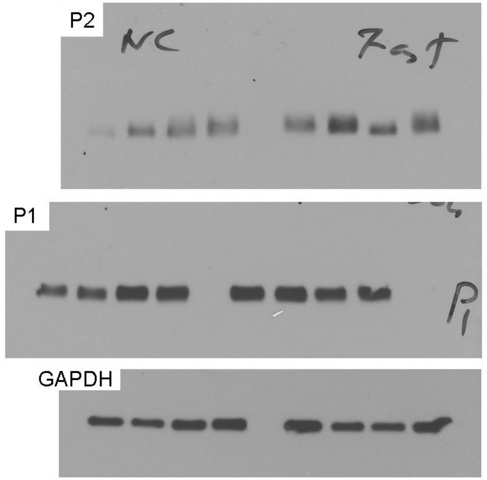

Fig. 2k

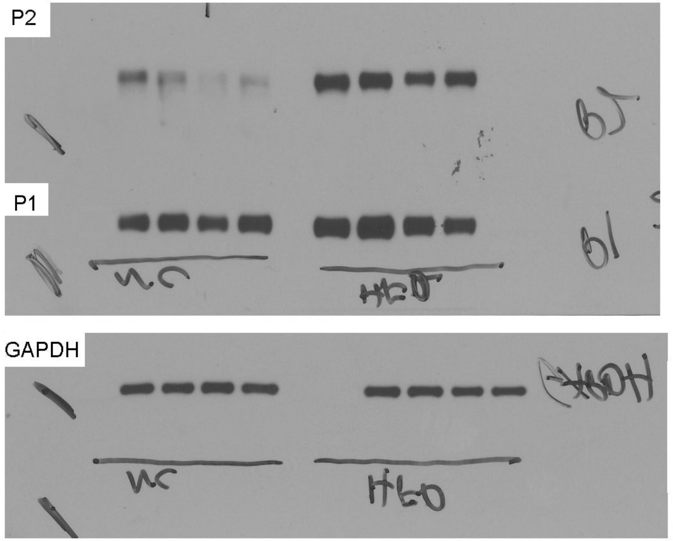

Fig. 2l

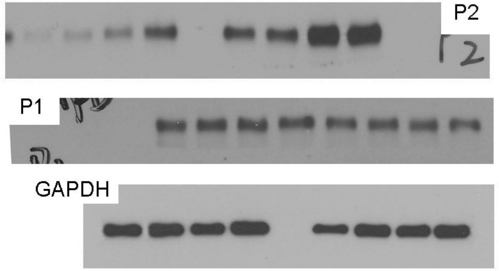

Fig. 2m

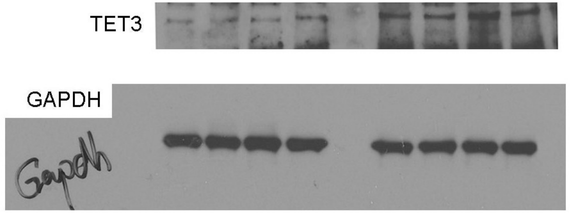

Fig. 2o

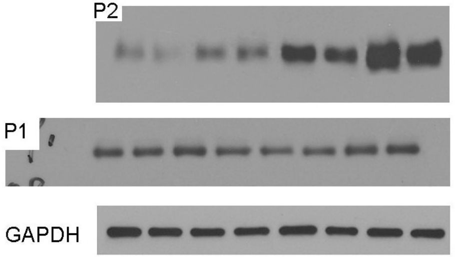

Fig. 2p

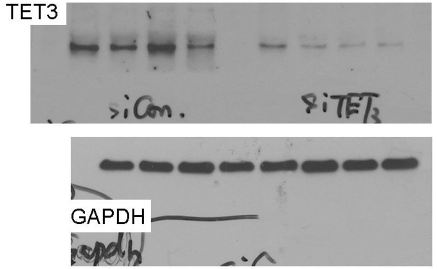

Fig. 2r

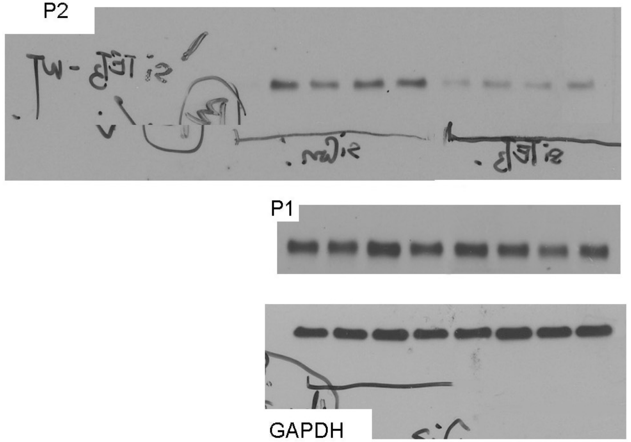

Fig. 3h

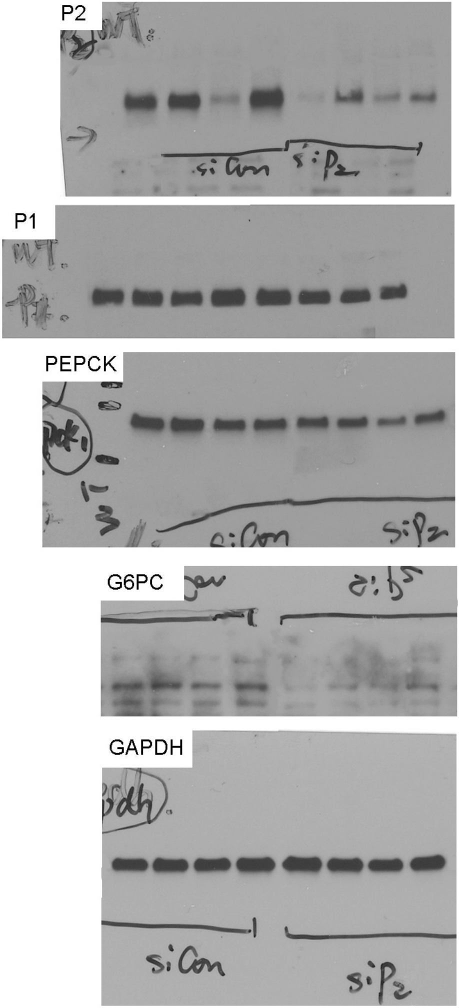

Fig. 5b

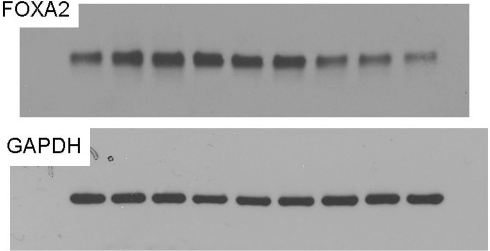

Fig. 5c

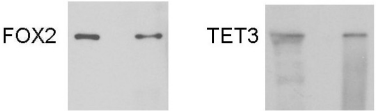

Fig. 5e

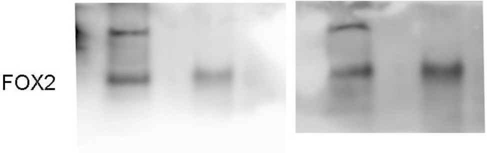

Fig. 6c left

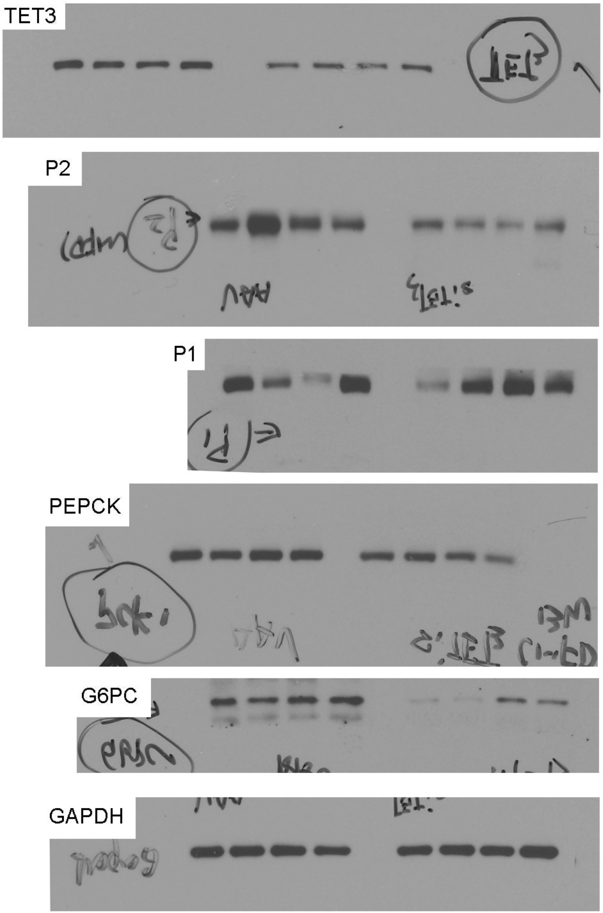

Fig. 6c right

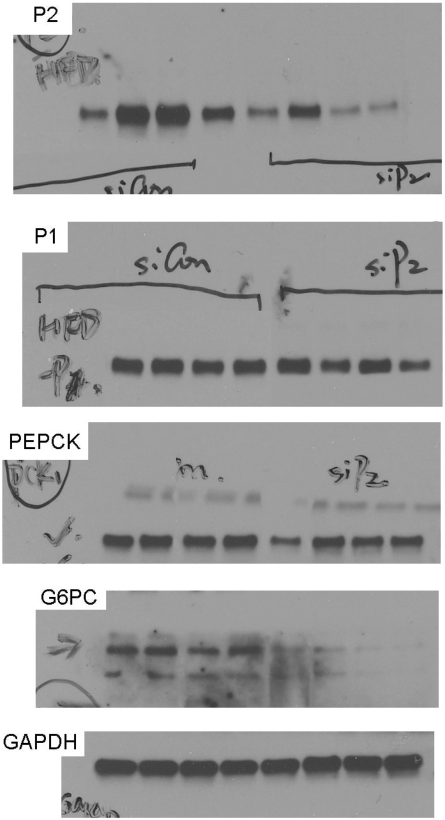

S 3d

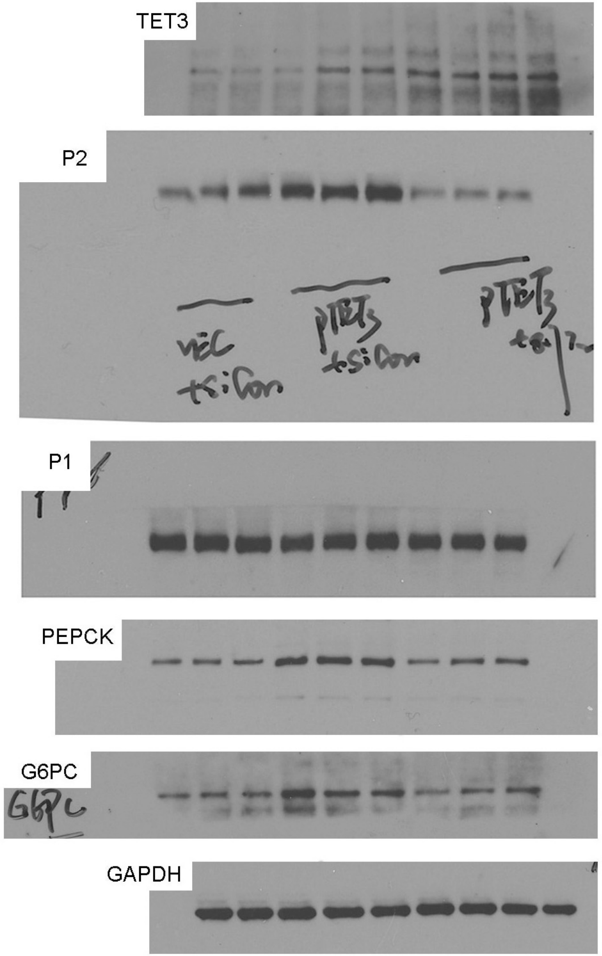
TET3
P2
P1
PEPCK
G6PC
GAPDH

S 6d

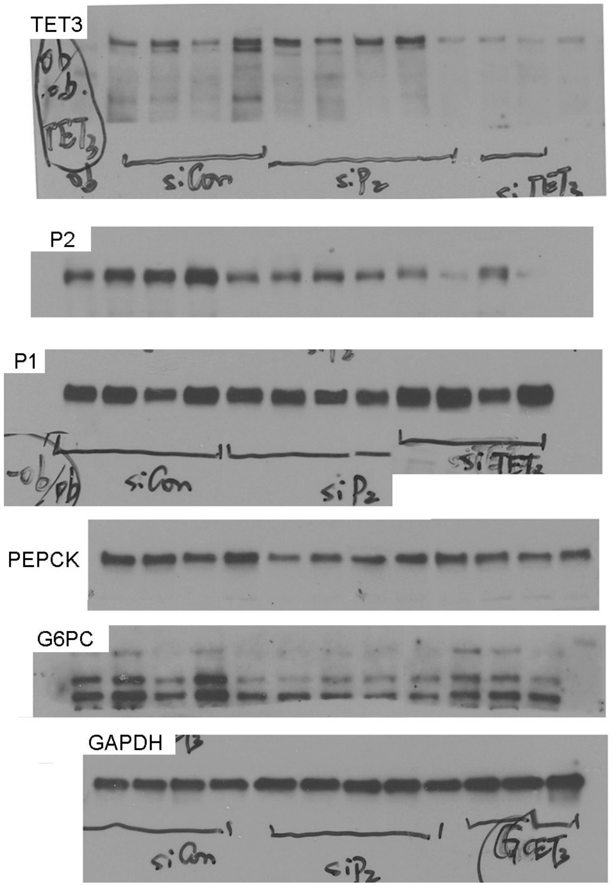
TET3
P2
P1
PEPCK
G6PC
GAPDH

S 5b

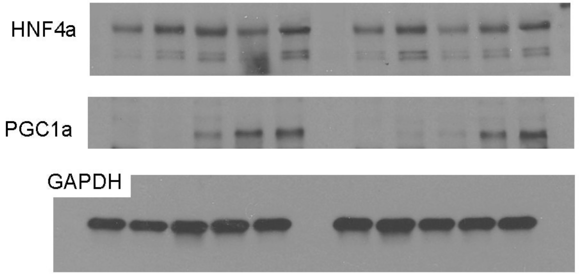
HNF4a
PGC1a
GAPDH
